# Differential gene expression in non-transgenic and transgenic “M.26” apple overexpressing a peach CBF gene during the transition from eco-dormancy to bud break

**DOI:** 10.1038/s41438-019-0168-9

**Published:** 2019-07-11

**Authors:** Timothy Artlip, Adam McDermaid, Qin Ma, Michael Wisniewski

**Affiliations:** 1 USDA-ARS-Appalachian Fruit Research Station, Kearneysville, WV 25430 USA; 2 Agronomy, Horticulture & Plant Science, South Dakota State University, Brookings, SD 57007 USA; 3 Present Address: Imagenetics, Sanford Health, Sioux Falls, SD 57007 USA; 4 Present Address: SBS-Biomedical Informatics, The Ohio State University, Columbus, OH 43210 USA

**Keywords:** Plant molecular biology, Abiotic

## Abstract

The CBF signal pathway is responsible for a significant portion of plant responses to low temperature and freezing. Overexpression of *CBF* genes in model organisms such as *Arabidopsis thaliana* enhances abiotic stress tolerance but also reduces growth. In addition to these effects, overexpression of the peach (*Prunus persica* [L.] Batsch) *CBF1* gene in transgenic apple (*Malus* x *domestica* Borkh.) line T166 also results in early entry into and late exit from dormancy. Although the regulation of dormancy-induction and dormancy-release occur while the *CBF* regulon is operative in perennial, woody plants, how overexpression of *CBF1* affects these dormancy-related changes in gene expression is incompletely understood. The objective of the present study was to characterize global changes in gene expression in peach *CBF1*-overexpressing and non-transformed apple bark tissues at different states of dormancy via RNA-seq. RNA-seq bioinformatics data was confirmed by RT-qPCR on a number of genes. Results indicate that the greatest number of significantly differentially expressed genes (DEGs) occurred in April when dormancy release and bud break normally occur but are delayed in Line T166. Genes involved in storage and inactivation of auxin, GA, and cytokinin were generally upregulated in T166 in April, while those for biosynthesis, uptake or signal transduction were generally downregulated in T166. Genes for cell division and cambial growth were also downregulated in T166 relative to the non-transformed line. These data suggest that overexpression of the peach *CBF1* gene impacts growth hormone homeostasis and as a result the activation of growth in the spring, and most likely growth cessation in the fall as well.

## Introduction

The domesticated apple, *Malus* × *domestica* Borkh., is a member of the family Rosaceae, tribe Pyreae, and is grown world-wide. Over 100 varieties are grown commercially in the United States, with an annual average of 240 million bushels of apples worth close to $4 billion (wholesale) (http://usapple.org/). Global production of apples for 2016/2017 exceeded 77 million metric tons (https://www.statista.com/statistics/279555/global-top-apple-producing-countries/).

Erratic weather patterns, as a symptom of climate change, pose an increasing threat to industry profitability. Data from the USDA Risk Management Agency for the years 2007–2017 indicates that apple growers claimed $157,177,390 in insured losses from freeze damage (https://www.rma.usda.gov/informationtools/). The majority of this damage occurred in the spring, when abnormally warm temperatures were followed by freezing temperatures^1^. For example, spring frost events in 2007 and 2017 each resulted in $1 billion in losses from all crops^2^ (https://www.ncdc.noaa.gov/billions/events/US/1980-2017). During these events, unseasonably warm temperatures in early spring (late February–March) resulted in the deacclimation of ecodormant buds and early bud break in many temperate, perennial fruit crops. The occurrence of subsequent freezing temperatures several weeks later (April) when flowers and early vegetative growth with little freezing tolerance were present resulted in high levels of frost injury^3^.

While dormancy and cold hardiness in perennial, woody plants are closely related, they each exhibit distinct regulatory aspects and phenology. Dormancy progresses seasonally through several phases regulated by both intrinsic (paradormancy and endodormancy) and extrinsic (ecodormancy) factors^4^. While growth during paradormancy and endodormancy is arrested by specific endogenous signals from within the plant, regulation of growth during ecodormancy is regulated by temperature and/or day length. Collectively, entry into and exit from dormancy represents a dynamic process involving both hereditary and epigenetic regulated changes in gene expression^5–8^.

Cold acclimation, the ability to tolerate freezing temperatures, is a complex and dynamic process. Low temperature survival depends on a combination of biophysical and biochemical factors, often driven by the upregulation or downregulation of specific sets of genes^9–12^. Among several other factors, research on cold acclimation has identified several COld-Responsive (COR) proteins, such as dehydrins, and transcription factors that control the expression of suites of *COR* genes, often referred to as a cold regulon. CBF/DREB transcription factors are integral to responses to low temperature and drought and are responsible for approximately 12–20% of cold-induced transcriptional changes in *Arabidopsis*^13,14^.

CBFs/DREBs are members of the AP2/ERF family of transcription factors that bind to a promoter motif consisting of A/GCCGAC, commonly referred to as the C-repeat or Drought Response Element. *CBF* genes have been identified in all investigated higher angiosperm species, including woody perennials. All CBF amino acid sequences feature common motifs in addition to the characteristic AP2 DNA binding domain^13^. The number of *CBF* genes in a genome varies from species to species, with some examples displaying the conserved motifs but not actually participating in low temperature responses. Apple and peach (*Prunus persica* [L.] Batsch) have five and six *CBF* genes, respectively^11^. The importance of *CBF* genes to cold hardiness has been demonstrated repeatedly in different plant systems^1^. Regulation of *CBF* genes is complex in *Arabidopsis*, with a variety of kinases, transcription factors, light- and photoperiod-related proteins, hormones, and degradation factors such as ubiquitin potentially playing roles^15^. Similar genes and proteins are found in apple, peach and other woody, perennial species suggesting that their *CBF* genes are regulated in a similar fashion^1,11^.

Wisniewski et al.^16^ reported that over-expression of a peach *CBF* gene (*PpCBF1*) in apple results in enhanced freezing tolerance, growth reduction, early onset of dormancy triggered by short days, and delayed bud break in the spring. The response to short days was especially novel, since growth cessation in apple is typically not impacted by short days^17^. These phenotypes were confirmed in a field planting maintained for three years^18^. Wisniewski et al.^19^ provided a model that suggested that CBF regulated cold acclimation, dormancy, and growth through *COR*, *DAM*, and *RGL* genes, respectively. It was also suggested that overexpression of *PpCBF1* regulated the expression of an apple *Early Bud Break* gene (*EBB*) that had been shown to regulate bud break in poplar^20^.

The growth-related and dormancy-related genes examined by Wisniewski et al.^19^, however, represent only a small number of the potential genes whose expression may be altered directly or indirectly by over-expression of the peach *CBF* gene in apple. Understanding complex pleiotropic effects requires studies of global gene transcription over time. In the current study, field samples of apple bark tissues were collected over several months from non-transgenic “M.26” apple trees and transgenic “M.26” apple trees overexpressing a peach *CBF* (*PpCBF1*) gene to provide a comprehensive overview of the differentially expressed genes (DEGs) associated with the phenotypes of these two genotypes from Ecodormancy (February, March, and April) to active growth (July), with an emphasis during bud break (April).

## Results

High-throughput sequencing resulted in 191.78 million high-quality, single-end reads (Table 1). An average of 89% of the clean reads were successfully aligned to the apple reference genome^21^, and an average of 31,483 genes or protein coding transcripts were identified between both genotypes and over all timepoints. This represents approximately 54.9% of the total predicted transcripts in the apple reference genome v1.0. Log_2_-transformation of the counts display similar distributions between the samples (Supplemental Fig. 1).

**Table 1:** Total RNA-seq reads from the T166 and “M.26” bark tissue samples

Sample	Genotype	Month	Replicate	Reads	Overall alignment (%)	Genes identified
1	M26	Feb	1	10,788,833	89.74	31,982
2	M26	Feb	2	8,194,111	90.65	30,761
3	M26	Feb	3	6,576,203	88.92	29,834
4	M26	Mar	1	7,113,401	89.29	29,874
5	M26	Mar	2	8,381,946	89.52	30,608
6	M26	Mar	3	8,751,294	90.12	30,712
7	M26	Apr	1	7,564,861	89.00	33,168
8	M26	Apr	2	8,398,890	89.18	33,147
9	M26	Apr	3	7,597,188	89.04	32,173
10	M26	Jul	1	8,013,981	88.03	33,558
11	M26	Jul	2	8,391,855	90.87	34,167
12	M26	Jul	3	8,111,513	90.75	33,732
13	T166	Feb	1	7,497,785	88.67	29,546
14	T166	Feb	2	8,400,121	89.25	30,119
15	T166	Feb	3	7,955,107	88.86	29,948
16	T166	Mar	1	8,862,755	88.44	30,172
17	T166	Mar	2	7,300,094	88.24	29,207
18	T166	Mar	3	7,088,792	88.60	28,988
19	T166	Apr	1	8,302,823	88.03	31,814
20	T166	Apr	2	6,878,138	86.27	30,711
21	T166	Apr	3	7,411,775	86.34	31,294
22	T166	Jul	1	8,358,582	89.16	33,605
23	T166	Jul	2	8,225,370	89.40	33,327
24	T166	Jul	3	7,617,277	88.91	33,149
Total	NA	NA	NA	191,782,695	89.02	31,483

Each genotype had three biological replicates per timepoint

Overall analysis of differentially upregulated and downregulated genes based on the experimental variables: (1) comparisons between the genotypes (transgenic vs. non-transgenic) at each of the sampled months; (2) genes that were differentially expressed over time within a genotype; (3) genes that were differentially expressed in one sampling point (month) to the next sampling point, and; (4) genes that were differentially expressed based on an interaction between genotype and time. Upregulation and downregulation was based on log_2_ fold-change >1 and log_2_ fold-change <−1, respectively, for genes with adjusted *p*-value < 0.05.

**Fig. 1:**
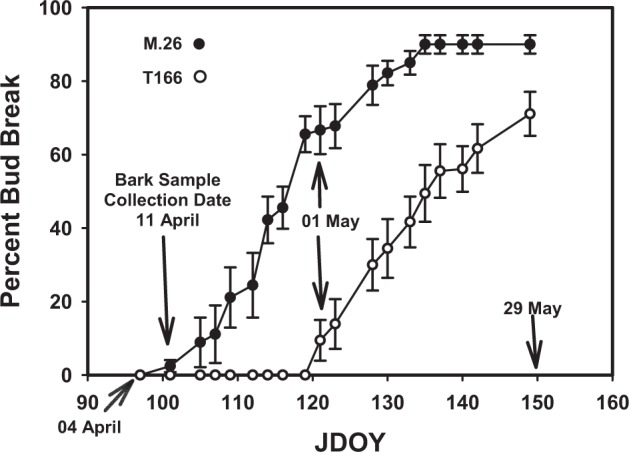
Bud break data for field-grown M.26 and T166 trees in 2013. JDOY is Julian Day of Year. Filled symbols, M.26. Open symbols, T166. Three shoots on each of three trees of M.26 and T166 were tagged and bud break from 20 individual lateral buds from the terminal bud were tracked. Percent bud break is mean ± s.d., *n* = 60

The four comparisons between the experimental variables provide different types of information. Comparison (1), “M.26” vs. T166 by month, gives a direct view of the differences in gene expression between the two genotypes at specific timepoints. The number of DEGs in the transgenic genotype (T166) during the latter months of winter (February and March) were similar, averaging about 1400 genes. Notably, there were a much greater number of downregulated genes in the February samples than in the March samples. The greatest number of DEGs between the two genotypes occurred in the April sampling with over 4000 DEGs. This result indicates that significant differences in gene expression between the transgenic (T166) and non-transgenic (“M.26”) exist at the time bud break was beginning in the “M.26” trees but not in the T166 trees (Fig. 1).

Comparison (2) indicates the effect of time on the expression level of genes within each genotype. Genes being identified as DEGs would be any that have expression levels that significantly changed (up or down) over time. Overall, the number of differentially expressed genes were similar in “M.26” (11,469) and T166 (11,097). This does not indicate similar expression patterns over time, only that a similar number of genes exhibited significant changes in expression over the sampled period (Feb–July).

Comparison (3) provides information on changes in gene expression from one sampling timepoint to the next within each genotype. One of the notable differences in this set of comparisons is in the number of DEGs in the Feb–Mar comparison for each genotype. The non-transgenic “M.26” genotype exhibited a much greater number of differentially expressed genes (2813) in this monthly comparison than the transgenic T166 genotype (530), indicating that “M.26” was undergoing a greater shift in gene expression than T166 in the late winter to early spring.

Comparison (4) indicates the number of genes who expression was significantly affected by the interaction between genotype and time. Genes identified as being DEGs in this comparison are those whose expression was significantly different between the two genotypes over the time course of sampling (Feb–Jul). These genes would be the most appropriate for further investigation, as their expression differs between the “M.26” and T166 genotypes over time.

**Fig. 2:**
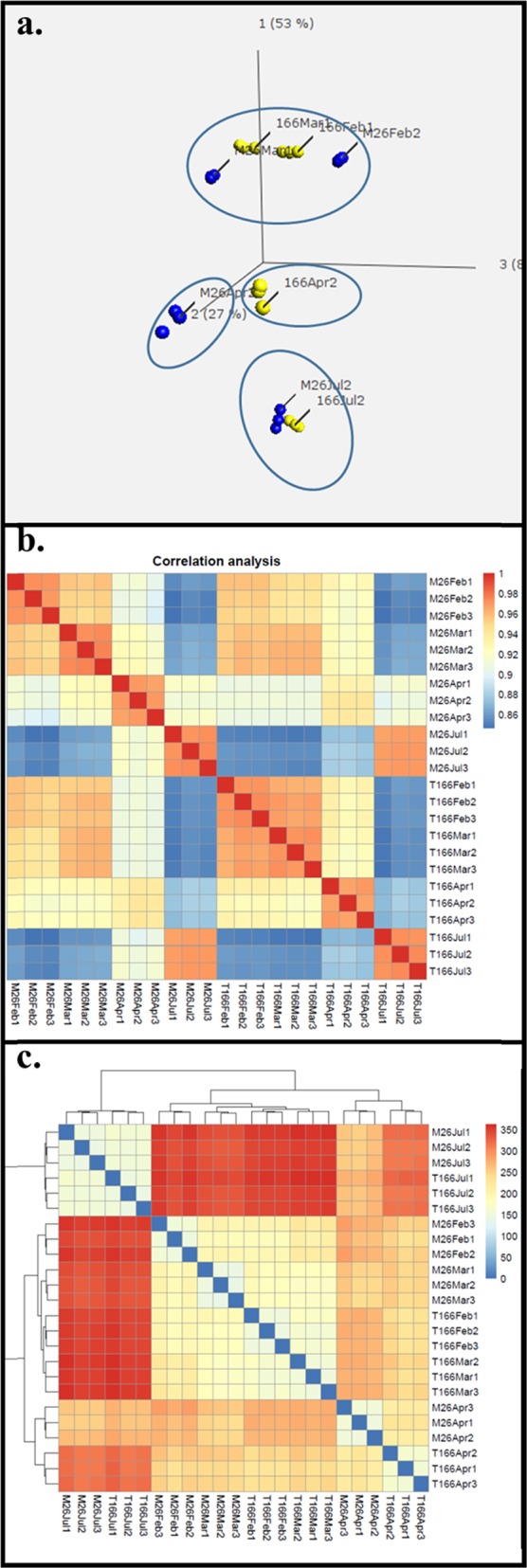
Graphical representations of Comparison 1 (genotype by month) data. **a** PCoA analysis. **b** Correlation-matrix: results of the correlation analysis displayed in matrix format. Each row and each column represents a single sample. Colored cells indicate the correlation value between the row and column sample based on the read count for each gene. Blue colors indicate lower correlation, and red colors indicate higher correlation. **c** Sample-distances: a sample distance matrix with accompanying clustering for each sample. Distances are calculated using a Euclidean distance, with larger distances (red) indicating more dissimilarity between the two samples in terms of genetic expression. Lower distances (blue) indicate more similar gene expression patterns, with identical expression patterns being represented by darker blue

The data depicted in comparison 1 (Table 2) can also be depicted by Principal Coordinate Analysis (PCoA, Fig. 2a), a correlation matrix (Fig. 2b), and a sample distance matrix (Fig. 2c). A PCoA analysis of DEGs in “M.26” vs. T166 was conducted using Qlucore software (v. 3.2). As illustrated in Fig. 2a, gene expression in T166 (yellow dots) clustered together in February and March and more distantly with the “M.26” samples (blue dots) from February and March. In fact, a finer grouping of the clusters indicates that both the “M.26” and T166 samples in February and March cluster separately from each other. The separation of “M.26” and T166 is most evident in the April samples where the two genotypes clustered independent of each other while the individual biological replicates within each genotype clustered together. Both genotypes clustered together with each other in the July samples, indicating that there were only minor differences in gene expression between the two groups.

**Table 2:** Upregulated and downregulated gene count comparisons of T66 vs. “M.26”

Comparison			Upregulated	Downregulated	Total
(1) M26 vs. T166	February		498	1189	1687
	March		734	384	1118
	April		1834	2177	4011
	July		146	56	202
(2) Time main effect	M26		7075	4394	11,469
	T166		7090	4007	11,097
(3) Month-to-Month	M26	Feb–Mar	1004	1809	2813
		Mar–Apr	3399	2336	5735
		Apr–Jul	3906	2043	5949
	T166	Feb–Mar	255	275	530
		Mar–Apr	3096	2419	5515
		Apr–Jul	5918	4225	10,143
(4) Time–strain interaction			1888	895	2783

Comparisons include genotype–genotype, time main effect, month-to-month by genotype, and time–genotype interaction comparisons. Upregulated and downregulated gene counts are provided based on log_2_ fold-change >1 and log_2_ fold-change *p*-value < 0.05

The correlation matrix (Fig. 2b) provides a picture of the similarity of the samples, with the color spectrum indicating how similar/dissimilar each sample is to another. The darker the red, the more directly similar the samples are, with the darkest red indicating a Pearson correlation of 1. Conversely, the blue cells indicate samples that are very dissimilar. In general, the results confirm the results obtained in the PCoA. For example, July “M.26” samples are similar to July T166 samples and both are highly dissimilar to February and March samples of both genotypes. Furthermore, April T166 and “M.26” samples are only slightly similar to each other, as was evident in the PCoA. The sample distance matrix figure (Fig. 2c) is comparable to the correlation matrix in that it shows how similar any two samples are. The distance metric used here is a basic Euclidean distance. Red indicates a more dissimilar pair of samples and blue indicates a more similar value. The main difference between the analyses in Fig. 2a–c is the metric used to determine similarity. Similar trends emerge, however, in each figure. Gene expression in “M.26” and T166 is most similar in July, less so in February and March, and most dissimilar in April. These data suggest that overexpression of *PpCBF1* has many pleiotropic effects during winter and early spring, and these downstream effects are minimized during summer.

**Fig. 3:**
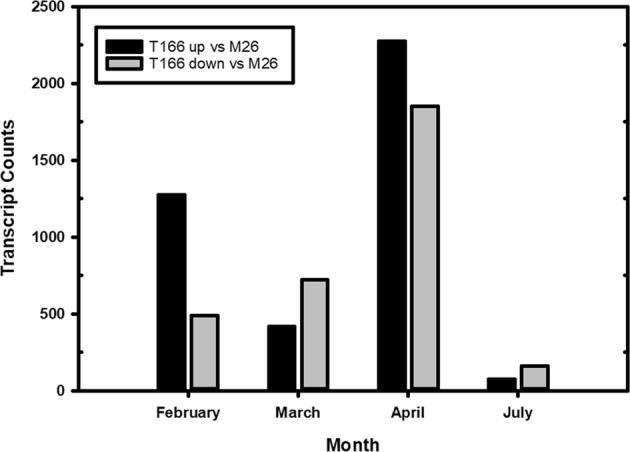
DGE-overview. Bar plots showing the number of upregulated and downregulated differentially expressed genes for the monthly comparison between T166 and “M.26” genotypes. The black bars represent the upregulated genes, i.e., genes with an adjusted *p*-value ≤ 0.05 and log fold-change ≥ 1. The grey bars represent the number of downregulated genes, i.e., genes with an adjusted *p*-value ≤ 0.05 and log fold-change ≤ −1. Upregulation and downregulation of genes is based on the change from T166 to “M.26”. Upregulated would indicate T166 has a higher average expression than “M.26” and lower for downregulated

How “M.26” and T166 compare in terms of upregulation and downregulation of gene expression is important to understanding the differences in dormancy and freezing tolerance in T166 relative to “M.26”. A complex pattern of differential gene expression was evident, with numerous genes being upregulated or downregulated in T166 trees relative to “M.26” trees (Fig. 3). The greatest disparity in the number of significantly upregulated and downregulated DEGs between T166 and M.26 occurred in February, followed by April. The number of significantly upregulated DEGs greatly exceeded the number of downregulated DEGs in those months. Conversely, the number of significantly downregulated DEGs exceeded the number of upregulated DEGs in March and July. The low number of DEGs in either direction in July suggests that few differences exists between T166 and “M.26” trees at that time of year.

**Fig. 4:**
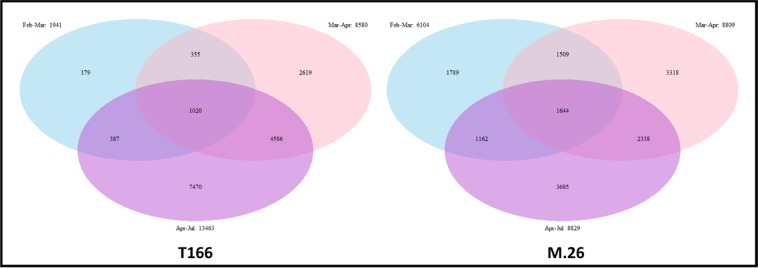
Venn diagrams of the changes in differentially expressed genes in T166 and “M.26” over time. T166 lags behind “M.26” gene expression changes in late winter—spring

The Venn diagrams (Fig. 4) of month to month differences in the number of unique DEGs within each genotype indicate important differences in the level of gene expression that may reflect differences in the timing at which growth was activated in the two genotypes. There were only a relatively small number of DEGs (179) in the Feb–March comparison in T166, while the “M.26” genotype exhibited 1789 unique DEGs in the same comparison. Such a high number of unique DEGs was not observed in the T166 trees until the comparison between Mar and April samples, suggesting that the activation of growth, as reflected in levels of DEGs, occurred later in T166 than it did in “M.26” trees.

**Fig. 5:**
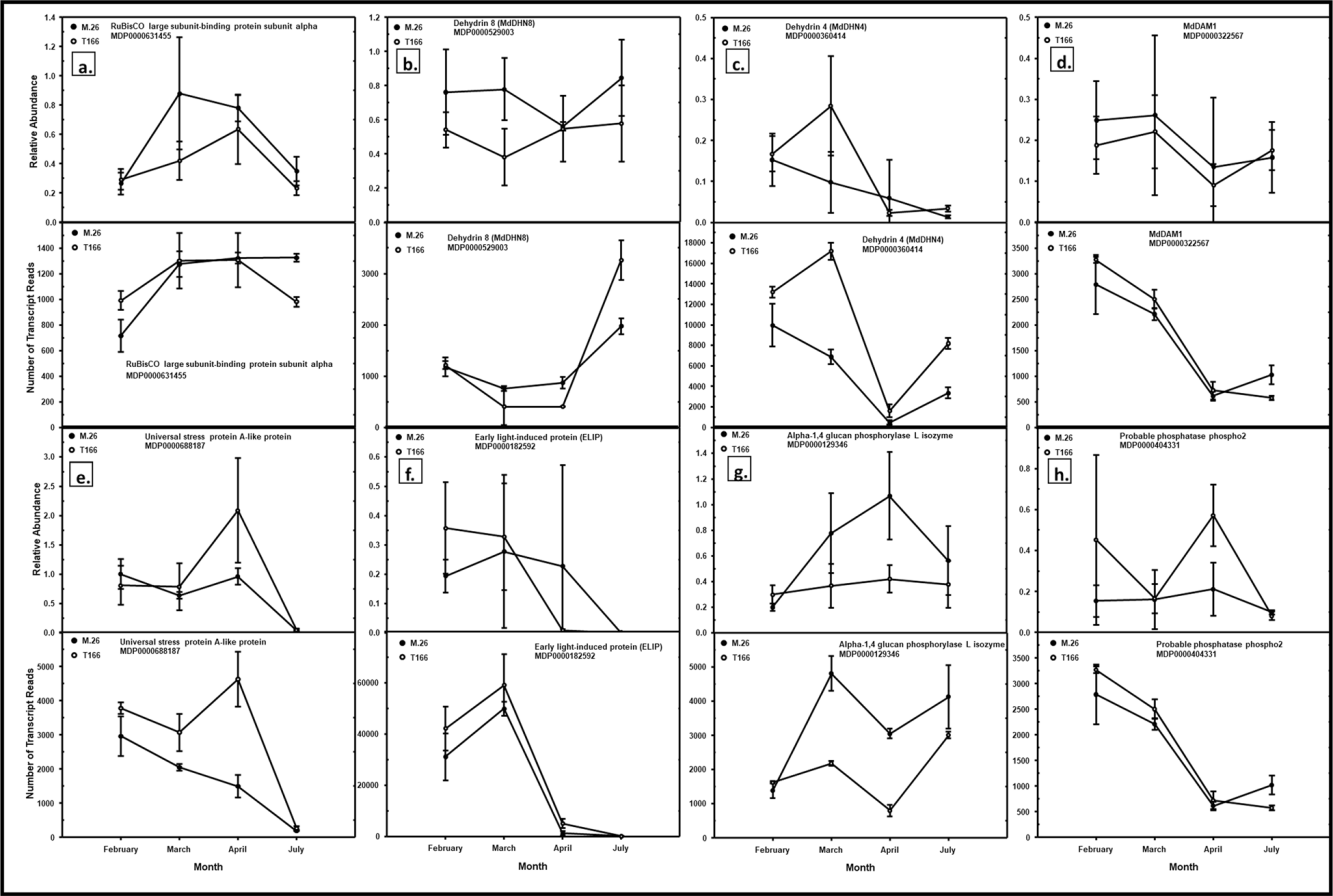
Model validation of the bioinformatics pipeline, with RT-qPCR and raw reads. RT-qPCR (top panels of each lettered pair) was conducted on selected genes to validate the results obtained by the RNA-Seq (bottom panels of each lettered pair). Solid circles, “M.26”. Open circles, T166. **a** RuBisCO large subunit-binding protein subunit alpha. **b** Dehydrin8 (MdDHN8). **c** Dehydrin4 (MdDHN4). **d** Dormancy-associated MADS-box1 (MdDAM1). **e** Universal stress protein A. **f** Early light-induced protein (ELIP). **g** α-1,4 glucan phosphorylase L isozyme. **h** Probable phosphatase phosphor 2. Means ± sd. *n* = 9 for RT-qPCR (3 biological replicates x 3 technical replicates; *n* = 3 for raw reads (3 biological replicates). Relative abundance for RT-qPCR graphs is abundance relative to the LTL1 endogenous reference gene, deemed as most stable across time by the NormFinder software^71^

Eight genes were chosen to validate the model by RT-qPCR (Fig. 5). The relative expression levels determined by RT-qPCR over time are comparable with the raw RNA-seq reads for the same samples. These trends can be compared to Base Mean data in Table 3. The base mean represents the average expression of that gene over both genotypes at a particular timepoint. The log_2_ fold change is then calculated to assess difference of individual genotype means from the base mean. The Wald test approach, which performs a parametric significance test of the selected factor level uses a negative binomial distribution. Significant *p*-values result from the factor being determined as significant in the Wald test. A positive log_2_ fold change indicates highly differential expression of the gene in T166 over that seen in “M.26”, while a negative log_2_ fold change indicates the converse—highly differential expression of the gene in T166 lower than that seen in “M.26”. The trends seen in Table 3 correspond to that seen in Fig. 4, thus validating the overall model for calling highly differentially expressed genes.

**Table 3:** Base mean and Log2 fold change data of genes used in RT-qPCR bioinformatics pipeline validation

Gene description	MDP model	Feb BM	Feb l2FC	Mar BM	Mar l2FC	Apr BM	Apr l2FC	Jul BM	Jul l2FC
Universal stress protein A-like protein	MDP0000688187	3319.822	0.432	2575.369	0.817	3196.586	1.965		
MdDHN4	MDP0000360414	11,386.714	0.489	12,444.817	1.591	1064.200	2.117	5807.932	1.405
MADS-box protein AGL24	MDP0000322567	2980.377	0.318	2356.479	0.427	659.739	0.561	780.211	−0.714
Alpha-1,4 glucan phosphorylase L isozyme	MDP0000129346	1487.964	0.298	3354.163	−0.880	1768.988	−1.603		
MdDHN8/ COR47	MDP0000529003			814.447	0.445	604.875	−0.761	2615.074	0.837
RuBisCO large subunit-binding protein subunit alpha	MDP0000631455	839.777	0.540	1271.574	0.271	1294.958	0.320	1136.768	−0.320
Early light-induced protein, chloroplastic	MDP0000182592	35,671.891	0.523	54,225.772	0.470	3232.784	2.359		
Probable phosphatase phospho2	MDP0000404331	178.894	1.257	143.378	2.306	301.816	3.230		

The data can be compared to those presented in Fig. 5. BM, base mean, which represents the average expression of that gene over both genotypes. L2FC, log2 fold change of T166 relative to “M.26”. Missing data indicates that the gene was not significantly differentially expressed between T166 and “M.26”

Constitutive overexpression of the peach *CBF1* gene in T166 would be expected to result in the upregulation of downstream, stress-associated DEGs. In fact, a large number *Dehydrin* genes were highly upregulated in T166 vs. “M.26” in all months (Supplemental Table 2, Supplemental Figs. 2–4). Additionally, several *LEA* homologs, salt tolerance genes, two *superoxide dismutase* (*SOD*) genes, a cold acclimation *WCOR413-like* gene, and eleven out of twelve genes encoding a Universal Stress Protein domain were also upregulated in T166 compared to “M.26” in April. Two *Defender Against Cell Death* genes, involved in programmed cell death (PCD), were slightly upregulated in T166 relative to “M.26” in April. Sixty heat shock proteins (HSPs), HSP cognates, or heat stress transcription factors were identified among the expressed genes. Most of the small *HSP* genes were slightly to strongly upregulated in T166, while the majority of large *HSP* genes were downregulated in April. The heat-stress transcription factors were also largely upregulated in April. Numerous genes associated with redox reactions or responses to reactive oxygen species (ROS) were upregulated in T166, except for ascorbate oxidases, which were all downregulated in T166 relative to “M.26”. A majority of genes associated with biotic stress were downregulated in T166 relative to “M.26” in April. Eight of nine wound-induced or wound-responsive genes were upregulated, along with two defense-related genes in T166 compared to “M.26” in April. In like manner, seven probable WRKY transcription factors were also upregulated in T166 relative to “M.26” in April. In contrast, pathogenesis-related (PR) group 5 genes encoding *thaumatin-like* and *osmotin-like* proteins were largely downregulated. Thirteen *Senescence Associated Genes* (SAGs) were found to be highly upregulated in T166 relative to “M.26” in April. Twelve disease resistance genes were also identified that exhibited patterns of both upregulation and downregulation. Native *CBF* genes were not significantly differentially expressed in the April samples of T166 compared to “M.26”.

Even though the greatest disparity in the number significantly upregulated and downregulated DEGs between T166 and M.26 was observed in February, April samples are of particular importance due to the fact that “M.26” begins to exhibit bud break at that time, while buds inT166 trees are still ecodormant (Fig. 1)^18^. Most of the genes coding for cell division cycle proteins were downregulated in T166 compared to “M.26” in the April samples, including cyclins, CDKs, and all G2/ mitotic-specific cyclins (Supplemental Table 3; Supplemental Figs. 2 and 3). Nearly all the identified kinesin genes were downregulated as was an *LFR* (*LEAF AND FLOWER RELATED*) gene involved in leaf and flower development. Eleven genes encoding *Expansins* were all found to be downregulated in T166 compared to “M.26”.

Only a limited number of signal transduction pathway genes for growth-associated hormones (auxins, cytokinins, and gibberellins) were differentially expressed in April, mainly auxin-related and GA-related (Supplemental Table 4), and these were largely downregulated in T166 relative to “M.26”, including auxin influx and efflux carrier genes. In contrast, genes for inactivation by glycosylation or storage pathways were upregulated. These data are consistent with ecodormancy and the lack of bud break or growth observed in T166 trees compared to “M.26” trees^16,18,19^.

Abscisic acid (ABA) and ethylene, two inhibitory or senescence-related hormones, would be expected to perhaps exhibit some degree of upregulation in the T166 genotype relative to the “M.26” genotype given that bud break is not readily evident in T166 trees in April. In particular, biosynthetic or signal transduction pathways for these hormones might be upregulated in T166 relative to “M.26”. Instead, the pattern of gene expression for these pathways was inconsistent for both hormones (Supplemental Table 5). This includes genes encoding *Phospholipase D alpha 1* and *Phospholipase D delta 1*, which are involved in signal transduction pathways for both ABA and ethylene.

Numerous genes, other than those associated with hormone biosynthetic or signal transduction pathways, are associated with dormancy and growth. Therefore, the expression of genes reported in models for bud break, vernalization, and floral initiation were examined, recognizing that these processes may differ in herbaceous and woody perennials. The upregulation and downregulation of different light signal transduction pathway genes varied in T166 relative to “M.26” (Supplemental Table 6; Supplemental Figs. 2 and 3). Two *PHYB* genes exhibited contrasting patterns of expression, while both *PHYC* genes were upregulated in T166 relative to “M.26”. In contrast, a *PIF1* homolog and two *PIF3* homologs were all downregulated in T166 compared to “M.26”. Circadian clock genes (*REVILLE*-like, *TIC*, *XAP5 CIRCADIAN TIMEKEEPER*, *PFT1*, and *LHY*) had no consistent upregulated or downregulated expression trends in T166 relative to “M.26”. Similarly, genes identified with the autonomous and vernalization pathways were not uniformly differentially upregulated or downregulated in T166 relative to “M.26”. Partial homologs of the target of these pathways, *FLC*, exist in apple. Differentially expressed *Agamous-like (AGL) MADS-box* genes were identified, one of which was identified as *FLC-*like. The *FLC*-like gene was differentially downregulated in April in T166 relative to “M.26”. Two of these *MADS-box*/ *AGL* DEGs were previously identified as *Dormancy Associated MADS-box* (*DAM*) homologs, *MdDAM1* and *MdDAM2*^19^. *MdDAM1* was highly upregulated in T166 compared to “M.26” in early spring, but down-regulated in summer. In contrast, *MdDAM2* was differentially expressed only during bud break, again in T166 relative to “M.26”.

Vegetative or vascular growth initiation genes, such as *SOC*, and *ANT*/ *AIL1*, were found to be highly differentially expressed, with *SOC* upregulated and *ANT/AIL1* downregulated between T166 and “M.26”, respectively (Supplemental Table 6; Supplemental Figs. 2 and 3), but in opposite directions. Four *CONSTANS* (*CO*)—like homologs were upregulated in T166 compared to “M.26”, but all the gene sequences were poor matches to either the *Arabidopsis* or *Populus* versions. Nine *Squamosa promoter-binding-like* (*SPL*) protein genes were highly differentially expressed, with only one being upregulated in T166 relative to “M.26”. Five *CLAVATA1* genes, associated with meristem and floral initiation in *Arabidopsis*, were downregulated in T166 vs. “M.26”. No evidence of differential expression patterns was observed for *FT* and *TFL*. These genes are typically expressed in buds^22,23^, so it is not surprising that expression, differential or otherwise, was not detected since bark tissues were examined in the current study. The gene encoding the PXY receptor, associated with cambial cell division^24^, was strongly downregulated in T166 relative to “M.26” in both Feb and April. In contrast, several *knotted-1-like* (*KNAP*) genes, also involved with cambial cell division, were upregulated in T166 relative to “M.26”.

The expression of genes associated with the plasmodesmata (PD) hypothesis of dormancy regulation^25^ were also examined. This hypothesis suggests that PD sphincters and their degradation regulate the onset and release of dormancy in buds (Supplemental Table 7). All *callose synthase* genes and most *Glucan endo-1,3-beta-glucosidase* (glucan hydrolase group 17) genes were downregulated in T166 relative to M.26. Five *remorin* family genes were detected, with a heterogeneous expression pattern.

## Discussion

The role of CBF transcription factors in low temperature/freezing and water deficit responses has been well documented in a variety of plant families and genera, including woody perennials^1,13,26,27^. Numerous abiotic-stress-responsive genes possessing the canonical A/GCCAG core motif in their promoters have been shown to be downstream targets of CBF binding^28,29^. Studies in plants overexpressing *CBF* genes have demonstrated increased survivability under artificial and natural conditions of freezing or water limitation^11,13,26,27^. These studies reported an improvement of −2 to −5 °C in freezing tolerance depending on the plant system. Wisniewski et al.^16^ reported increases of −3 to −4 °C in both non-cold-acclimated and cold-acclimated greenhouse-grown apple trees overexpressing the peach *CBF1* gene (*PpCBF1*, line T166). This effect was later confirmed in field studies, where non-acclimated trees in mid-summer had similar increases in freezing tolerance^18^. Artlip et al.^30^ reported that the effect on cold hardiness was not graft transmissible through a transgenic T166 rootstock, but juvenility (time to flowering) was affected in non-transgenic scions grafted to transgenic T166 rootstock.

Plant processes other than stress tolerance have been reported to be improved by *CBF* overexpression. Constitutive overexpression of *CBF* genes has been reported to result in diminished growth in both herbaceous and woody plant systems, including apple^16,18,19^. Overexpression of peach *CBF1* in apple also affects entry into and exit from dormancy. Wisniewski et al.^16^, Artlip et al.^18^, and Wisniewski et al.^19^ demonstrated that apple gained a novel sensitivity to short day (SD) photoperiods, manifested by early leaf senescence and bud set. Several genes known to participate in dormancy processes were examined by Wisniewski et al.^19^. They reported a correlation between gene expression patterns in spring and the bud break patterns exhibited by M.26 and T166. While Wisniewski et al.^19^ provided information on the expression of a few genes related to growth and dormancy, a more complete analysis is provided in the present study. This information provides a more comprehensive picture of how the peach *CBF1* gene affects so many physiological processes and pathways, especially those related to dormancy and growth. Samples taken from trees grown in the field provides an excellent measure of the impact of CBF proteins in a natural setting.

Based on the DEGs identified in the present study it appears that the overexpression of *CBF* in apple impacts gene expression in a complex manner that potentially impacts numerous different processes and pathways. The RNA-seq analysis conducted in the present study detected approximately 46,500 genes constituting ≈73% of the predicted apple genome (Table 1). While there is considerable overlap of the genes being expressed between T166 and “M.26” during any given month (Supplemental Figs. 5–12), a significant level of difference was also detected (Figs. 3, 4, 5). PCoA indicated that gene expression in the July samples of the two genotypes was much more similar to each other than it was to gene expression in other months. It is likely that the effect of *PpCBF1* overexpression on gene expression is at a minimum during the summer months when tree growth and general metabolism are extremely active. Similarly, clustering or the relatedness of Feb and Mar samples for both genotypes, when growth processes are at a near minimum, were somewhat similar to each other and are distinctly separated from the Apr samples of both genotypes.

The Feb–Mar–Apr months, encompassing late winter and early spring, are a time of transition for perennial, woody plants in the northern hemisphere, as during that time they overcome ecodormancy and undergo bud break. A combination of photoperiod and temperature typically fosters extensive changes in gene expression in most perennial woody plants^1,6,31,32^. In contrast, apple (and pear) depend solely on temperature to induce entry and release from ecodormancy^17^. The novel sensitivity to short days reported by Wisniewski et al.^16^, Artlip et al.^18^, Wisniewski et al.^19^, and Artlip et al.^30^ are reflected in the DEGs that were identified in the current study.

Several types of stress-related protein genes were examined to determine if they were upregulated by overexpression of *PpCBF1*. *Dehydrin* and *Late Embyrogenesis Abundant* (*LEA*) genes were upregulated in T166, relative to the non-transgenic parent genotype “M.26”, as has been previously documented in other species. These genes typically contain a C-repeat/DRE binding site for CBF/DREBs and are recognized as targets of CBF/DREB proteins. The results of the RNA-seq analysis agree with Wisniewski et al.^16,19^. *DREB2A* genes were also upregulated in T166, along with the *CBF*/ *DREB1 Tiny* gene. It is possible that the latter genes may respond to multiple cues or have C-repeat/DREB sites in their promoters. Notably, no native *CBF* genes were differentially expressed, despite the presence of C-repeats in the promoters of *MdCBF1*, *2*, *3*, and *5*^19^. Other factors, however, also play a role in *CBF* expression and regulation^1,13,15^, and it is possible that regulation of apple *CBF* genes may differ in regard to the *Arabidopsis* model. Indeed, few of the *MdCBFx* promoters contain regulatory motifs consistent with Shi et al.^15^ or Wisniewski et al.^1^. Additional research is clearly required on how native *MdCBFx* expression is regulated.

Many additional stress proteins were identified as being up-regulated in T166. Both small (<30 kDa) and large (>70 kDa) *Heat Shock Protein* (*HSP*) genes were consistently up-regulated in T166 in Feb and Mar. *HSP*s are known to be induced by low temperatures^33^, and so *PpCBF1* overexpression may have contributed to the observed upregulation. In April samples of T166 the upregulation of small *HSP*s was maintained while large *HSP*s were largely downregulated. Concurrently, several *HSP* transcription factors were also upregulated in April samples, suggesting that these may primarily regulate small *HSP* gene expression. Numerous genes encoding proteins with a bacterial Universal Stress Protein motif were also differentially expressed, generally upregulated in T166 compared to “M.26”. As noted by Kerk et al.^34^, the functionality of these domains in *Arabidopsis thaliana* is uncertain. Thus, additional research is needed to discern what the functions of the identified proteins are in apple bark. Genes associated with redox or ROS homeostasis were inconsistent in overall trends, perhaps reflecting the relatively dormant state of T166. Transcripts for *osmotin*-like and *thaumatin*-like genes were decidedly downregulated in T166 relative to ‘M.26’ in the monthly samples. These ABA-responsive and wound-responsive proteins are members of the pathogenesis-related 5 (PR5) group of pathogen-response proteins^33^. Notably, many of other wound-induced and pathogen-induced genes were upregulated in T166, suggesting a complex interplay between CBF and signal transduction pathways responsible for the expression of defense-related genes.

Comparisons between T166 and “M.26” made in the current study indicate that large differences in gene expression exist at the time bud break begins to occur in “M.26” but not T166. Differences in the timing of bud break were also noted in Artlip et al.^18^, who reported that bud break in “M.26” was evident in about 5% trees at the time (April) the samples were taken for the RNA-seq study. In contrast bud break in T166 trees was not readily apparent until after an additional two weeks (see also Fig. 1, this study).

Wisniewski et al.^19^ examined a limited suite of genes associated with growth and dormancy (*RGL*/ *DELLA* genes, *MdDAM*s *1*-*3* and *MdEBB1*) in T166 and “M.26” trees and determined that some temporal differences in the level of expression of these genes were evident in these two genotypes. In the present study, a more comprehensive analysis of gene expression was conducted that could serve as a basis for understanding the effect of *CBF* overexpression on freezing tolerance and dormancy, as well as several other non-target parameters, such as growth and flowering.

Plant growth regulator (PGR) levels impact every aspect of plant growth and development, including dormancy. Growth-promoting PGRs such as auxins, gibberellins, and cytokinins have been reported to be affected by CBF overexpression^35–37^. Genes responsible for the storage or catabolism of auxins were largely upregulated in April samples of T166 compared to “M.26”, suggesting that auxin levels were low and thus may have inhibited the onset of growth in T166. Indeed, cambial transcriptomic and cambial cell dynamic studies in aspen strongly suggest that auxin transport and perception are vital to transitions from growth to dormancy and the converse^5,7,29,38^.

GA biosynthetic-enzyme encoded genes such as *ent*-kaurene oxidases are noticeably upregulated in T166 relative to “M.26”, however, several GA2 oxidase (catabolic) genes were also upregulated in T166 in Apr. Achard et al.^35^, Suo et al.^37^, and Niu et al.^36^ reported on changes in the expression levels of GA-biosynthetic and deactivating genes in plants over-expressing *CBF* genes in *Arabidopsis thaliana*, soybean (*Glycine max*), and tobacco (*Nicotiana tabacum*), respectively. Suo et al.^37^ and Niu et al.^36^ reported decreased GA levels in *CBF*-overexpressing plants. Low levels of GA stabilize growth-inhibiting RGL/DELLA proteins^39^, which can be exacerbated by *CBF* overexpression^35^. Transcripts from four *RGL*/ *DELLA* genes were detected, primarily in April, but were slightly downregulated in T166 relative to “M.26”. The expression of two of these genes, *RGL1a* and *1b*, was previously characterized by Wisniewski et al.^19^, who also found that expression level of these genes in April was not substantially different between T166 and “M.26”. Wisniewski et al.^19^, however, did find differences during other months, suggesting that those months may be critical to the impact of *CBF* overexpression on growth inhibition. Important GA-responsive genes may also be downregulated in T166, and thus contribute to reduced growth. For example, transcripts encoding numerous putative *GID1* GA-receptors were identified in the current transcriptome analysis and found to be significantly downregulated in T166. GID1 proteins bind to GA and foster the degradation of RGL/DELLA proteins, thus relieving RGL/DELLA growth inhibition^40^. Data from the current study, however, are equivocal in terms of evident expression patterns and the subject needs to be examined further.

Bhalerao and Fischer^7^ noted that reduced response to cytokinins can lead to reduced cambial cell division rates, and it is well known that cytokinin levels can also impact growth. Therefore, genes encoding cytokinin storage and inactivation enzymes were examined. In general, these genes were upregulated in T166 relative to “M.26”, suggesting another potential mechanism by which *PpCBF1* overexpression may negatively affect growth and development. Indeed, genes encoding ANT/AIL (ANTINTEGUMENTA/ AINTEGUMENTA-LIKE) and CYCD (D-type cyclins) are all downregulated in T166 relative to “M.26” in April. ANT/ AIL positively regulate CYCD, increasing cell division^7^.

Inhibitory PGR-related genes associated with ABA and ethylene were examined. Genes encoding multiple members of the ABA signal transduction pathway (PYL/ PYR receptors, SnRK2 and 3 homologs, mitogen protein kinase cascade, Phospholipase D) were found to be differentially expressed over the entire sampling period, particularly in April. There was no consistent trend in expression, however, suggesting that *PpCBF1* overexpression does not impact ABA signal transduction directly. The expression of ethylene-related genes was similarly inconsistent with no evident pattern within or between the two genotypes. While terminal biosynthetic enzyme *ACC oxidase* homologs were generally upregulated in April T166 samples, as well as in a few other months, ethylene receptor genes varied in their level of expression.

Numerous genes and models have been reported that couple PGRs with entry into and exit from dormancy. Models of the regulation of dormancy in woody perennials have been reported that incorporate aspects of herbaceous models^6,7,41–43^. The general pathway includes photoperiod perception by phytochromes A and B which interact with the circadian rhythm proteins (LHY, TIC, and TOC). CO/FT modules are then stimulated, leading to AP1 homolog activation, followed by ANT/AIL1, and resulting in the induction of vegetative growth. Rinne et al.^25^ proposed the plasmodesmata (PD) hypothesis, wherein callose sphincters close off the PD during autumn, preventing stimulatory PGRs from reaching the apical meristem. As daylength increases, GA-inducible 1,3-β-glucanases degrade the callose plug, allowing stimulatory PGRs to enter meristematic cells and induce bud break. Tylewicz et al.^44^ further refined this this hypothesis by reporting that dormancy onset is ABA-dependent, with photoperiod playing a role. ABA has also been implicated in pear flower bud endodormancy, as reported by Li et al.^45^. Apple and pear are closely related, so it is possible that they share this mechanism as well. Indeed, apple and pear have closely-related *DAM* genes and expression patterns in common as assessed in seasonal tissue collections and growth chamber experiments^19,28,46,47^.

DEGs that are included in data or models presented by Schrader et al.^5^, Ding and Nilsson^40^, Bhalerao and Fischer^7^, and Xing et al.^48^ were observed in the current study. In April, *PhyA* and *B* expression was slightly downregulated in T166 compared to “M.26”, as was *CRYPTOCHROME1*. *PhyC* genes were upregulated also but have not been implicated in current models of dormancy regulation. Other prominent members of the circadian regulatory module were either not differentially expressed (e.g., *TIC*, *CCA1*) or exhibit mixed expression (upregulated or downregulated differential expression in T166 relative to “M.26”). Mixed expression was also observed for *GI*, *CO*, the *FT* homolog *MdFT2*, and *ANT/ AIL1*. It is perhaps unsurprising that *GI*, *CO*, and *ANT/AIL* display expression in cambial tissues, as light perception and transmission has been observed in bud and vascular tissues^7,30,49^. However, the presence of *MdFT2* in cambial tissues is apparently novel. Previous reports in apple indicate that *MdFT2* is highly expressed in developing fruit^23^. Given the known role of *FT* in floral induction, Mimida et al.^50^ suggested that *MdFT2* could have roles in floral organs and fruit development. The present study suggests that *MdFT2* could have roles in early-season cambial growth dynamics as well. Autonomous and Vernalization pathway genes were inconsistently differentially expressed. These pathways are typically considered part of the dormancy/flowering cycle of herbaceous plants, but also function in perennial, woody flowering plants as well^48^. The primary target of the autonomous and vernalization pathways is *FLC*. An *FLC* paralog, termed *MdMAF2* and previously reported in buds^51^, was slightly down regulated in T166 relative to “M.26”. Kumar et al.^52^ disagree with the assignment of *MdMAF2* as an *FLC* paralog, however. Such disagreement, coupled with the low transcript number observed, renders the relevance of *MdMAF2* expression in bark, differential or not, murky at best.

Other genes associated with either vegetative or vascular growth such as *SOC*, *SPL*, and *CLAVATA*^5,7,23,53^ were varied in their expression as well. *PXY*, associated with cambial cell fate decisions^7^, was negatively DE in T166 relative to “M.26”. The thinner stem diameters reported for T166 compared to “M.26” by Artlip et al.^18^ may reflect this downregulated expression. General genes related to growth, such as cyclins, cell division control proteins, and expansins^5,7^ were generally found to be downregulated in T166 relative to “M.26”, over the course of the study, especially in April. This may reflect the overall downregulation of growth-stimulating PGRs, resulting in the late bud break and reduced growth observed in T166. Expression of the *knotted1-like* (*MdKNAP*) genes were generally upregulated in T166 relative to “M.26”. The *MdKNAP1* and *2* genes are known to be expressed during growth and development^54^ in apple stem internode tissue, but only *MdKNAP1* was differentially expressed, with upregulation in T166 in the present study. It is also known that *Populus knotted1* homologs can have overlapping, but discrete functions in these processes^55,56^. In depth examination of the apple *KNAP* genes in T166 and “M.26” is clearly warranted to clarify what roles they may play in dormancy and growth.

Genes related to the PD hypothesis were generally downregulated in T166 (Callose Synthases, remorins, glucan endo-1,3-beta-glucosidase (Group 17 glucanases). Downregulated glucan endo-1,3-beta-glucosidases could contribute to delayed dormancy in T166, as Rinne et al.^25^ reported a positive correlation between their expression and dormancy release.

Dormancy associated MADS-box (DAM) proteins have been reported to regulate dormancy in peach^57–59^ and implicated in other species. The expression of apple *DAM* genes has been reported on by several groups, including Wisniewski et al.^19^ in bark tissues as *MdDAM*s*1*-*3*, and by Wu et al.^47^ in apical buds as *MdDAMa*, *MdDAMc*, and *MdSVPb*, respectively. In the present study, *MdDAM1*/ *MdDAMa* was detected as being slightly, but statistically significantly, upregulated in T166 in Feb, Mar, and April. *MdDAM2*/ *MdDAMc* was upregulated in T166 relative to “M.26” only in April, with no statistically significant differential expression data from Feb, Mar, and July. A lack of statistically significant differential expression data does not imply a lack of expression in either T166 or “M.26”. Rather, the expression levels in both genotypes were so similar that they simply were filtered out of the bioinformatics pipeline used in this study. As such, *MdDAM2*/ *MdDAMc*, along with *MdDAM3*/ *MdSVPb*, were not differentially expressed during the other months examined in this study. The expression data observed in the present study generally agrees with data presented by Wisniewski et al.^19^ with little difference in the expression of these genes between T166 and “M.26” bark tissues. Wu et al.^47^ focused primarily on *MdDAM* and *MdSVP* expression in apical buds. Similarly, Falavigna et al.^60^ compiled data from these studies and others, creating a model of DAM expression in apical buds as a function of dormancy. Their model implies elevated expression of *MdDAM2*/ *MdDAMc* coincident with the end of the growth cycle, followed by elevated expression of *MdDAM1*/ *MdDAMa* as a component of endodormancy. Elevated expression of a third *DAM*, *MdDAMb* (not reported in this study), fosters ecodormancy; its decreased expression then heralds budbreak^60^. It is likely that *MdDAMb* is not a DEG in bark tissues, or was not a DEG at the sampling timepoint. Overall, the data and interpretations of Wu et al.^47^ and Falavigna et al.^60^ largely agree with those presented by Wisniewski et al.^19^ and the present study, which both focused on bark (cambial) tissues rather than buds. In addition, many of the findings of the current study reflect those presented by Schrader et al.^5^ in poplar, indicating that conserved cambial growth dynamics exist between two species from different taxonomic families.

**Fig. 6:**
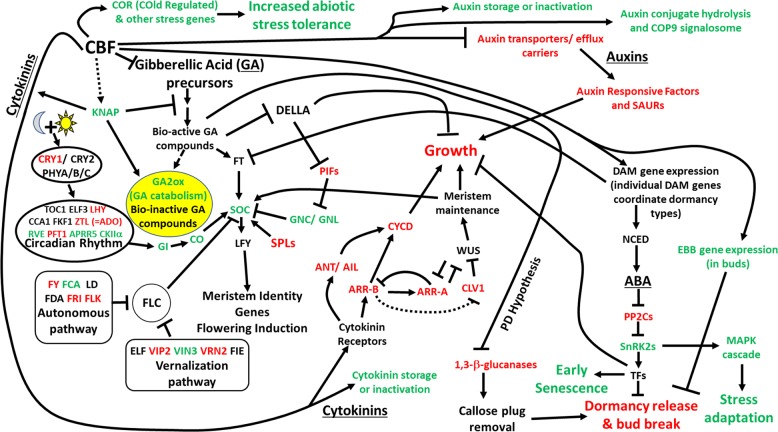
Potential interaction network of *CBF* overexpression effects in bark tissues primarily in April. Genes that are downregulated in T166 compared to M.26 are in red, while upregulated genes in T166 compared to M.26 are in green. Gene names in black indicate no difference between T166 and M.26 or non-uniform expression patterns of several gene family members. Solid lines indicate evidence in the literature or deduced in this study, dotted lines denote speculative interactions, arrowheads denote stimulatory actions, and **T** ends denote inhibitory actions. TFs, transcription factors; SAUR, Small auxin-up RNA; KNAP, Knotted1-likeAPple; CRY, cryptochrome; PHY, phytochrome; GI, GIgantea; CO, COnstans; SOC, suppressor of overexpression of constans 1; LFY, LeaFY; SPL, squamosa promoter binding like; PIF, phytochrome interacting factor; GNC/GNL, GATA Nitrate-inducible, Carbon-metabolism involved/GNC-like; CKIIα, Casein Kinase II alpha subunit; TOC, timing of CAB expression 1; LHY, late elongated HYpocotyl, CCA1, circadian and clock associated 1; ELF3, EarLy Flowering3; ZTL/ADO; Zeitlupe/Adagio; RVE, Reveille; PFT1, phytochrome and flowering integrator 1; FKF1, flavin-binding kelch repeat F box protein; FCA, FT, flowering time; FLC, flowering locus C; LD, LuminiDependens; FLK, flowering late KH motif; FRI, FRIgida; FCA, flowering control locus A; VRN2, vernalization2; VIN3, vernalization insensitive3; FIE, fertilization-independent endosperm; VIP3, Vernalization IndePendence 3; ANT/AIL, AiNTegumenta/AIntegumenta-Like; CYCD, Cyclin D Type; ARR-A/B, type-A/B/C *Arabidopsis* response regulator; CLV, Clavata; WUS, Wuschel; SnRK2, Sucrose Non-fermenting kinase 1 Related protein Kinase 2; PP2C, Mg2+-dependent and Mn2+-dependent serine-threonine phosphatases type 2C; NCED, 9-cisepoxycarotenoid Dioxygenase; EBB, Early Bud Break. Pathways adapted from information presented in the refs. ^19,20,25,30,39,40,44,48,55,60,72,73^ and at KEGG (https://www.genome.jp/dbget-bin/www_bget?pathway:mdm04016 and https://www.genome.jp/dbget-bin/www_bget?pathway:mdm04075). Data regarding EBB in buds taken from the ref. ^19^

Collectively, data in this study extend the findings reported by Wisniewski et al.^19^ and Artlip et al.^30^ A hypothetical framework of potential direct and indirect effects of *PpCBF1* overexpression is presented in Fig. 6. The current study indicates that the effect of *PpCBF1* overexpression in Line T166 is complex as evidenced by the significant differences in gene expression observed in the two genotypes in any given month (Supplemental Tables 2–7; Supplemental Figs. 5–12). *PpCBF1* overexpression appears to affect the biosynthesis, signal transduction, and inactivation of PGRs. The effect on PGR-related gene expression may then impact the complex process associated with bud break. In general, the changes in gene expression that were observed in “M.26” during the time of bud break and the re-activation of growth were somewhat delayed in T166. Closer examination of the genes identified in the present study is warranted to better understand the pleiotropic effects of *PpCBF1* overexpression in transgenic T166 apple.

## Materials and methods

### Plant material

Non-transgenic “M.26” and transgenic “M.26” (line T166) trees that were approximately three-years-old were used in this study. The T166 (PpCBF1-OX) line was initially described by Wisniewski et al.^16^. Briefly, M.26 leaves were subjected to *Agrobacterium*-mediated transformation with a vector consisting of a pBINPLUSARS backbone and the peach *PpCBF1* gene driven by a dual 35S enhancer segment derived from pRTL2. Plants were maintained in tissue culture, roots initiated, and plantlets established in growth chambers and greenhouse prior to being planted in October 2010 at the Appalachian Fruit Research Station, USDA-ARS, Kearneysville, WV per Artlip et al.^18^ Three trees each of “M.26” and T166 were used in this study as biological replicates.

Young, lateral branches were collected from “M.26” and T166 trees monthly in February, March, April, and July 2013. Bark tissues (cambium, phloem, and epidermis and/or phellem) were scraped from current year and one-year-old shoots and immediately placed in liquid nitrogen, lyophilized, and stored at −20 °C until use. Tissues from three trees of each genotype were sampled and maintained as independent biological replicates. Temperature and precipitation data were recorded daily during 2013. Trees had not been pruned during their initial three years of growth.

Dates of bud break for each tree were recorded during spring 2013. Percent bud break was determined thusly: three shoots on each of three trees of M.26 and T166 were tagged and bud break from 20 individual lateral buds from the terminal bud were tracked.

### RNA extraction, library construction, and sequencing

RNA was isolated from bark samples as described by Bai et al.^61^, with purification and library construction as previously described by Ballester et al.^62^. In brief, 1 ml Sarkosyl of 20% (w/v) was added to 10 ml of extraction buffer (2% CTAB, 2% polyvinylpyrrolidone (PVP) K-30 (soluble), 100 mM Tris HCl (pH 8.0), 25 mM EDTA, 2.0 M NaCl, 0.5 g/l spermidine (free acid) (HRS), 2% β-mercaptoethanol (added just before use). The extracted total RNA was dissolved in EB buffer (Qiagen, Germantown, MD) supplemented by 1× Ambion RNA secure (Invitrogen/Life Technologies, Carlsbad, CA). To activate RNA secure, the samples were incubated at 60 °C (in a water bath) for 10 min and then immediately put on ice. RNA quantity and quality were evaluated using a Nanodrop 1000 (Thermo Scientific, Waltham, MA). Immediately prior to mRNA isolation, RNA samples were treated with DNase I (amplification grade, Invitrogen) at 37 °C for 30 min followed by heat inactivation at 65 °C for 15 min. Each RNA sample was adjusted to contain 5 μg of total RNA. Library construction was performed using the protocol outlined in Zhong et al.^63^ and run in two lanes using the Illumina HiSeq2000 platform to obtain 51-bp single-end reads. Libraries from three independent biological replicates of each genotype at each timepoint were sequenced and analyzed.

### Bioinformatic analyses

The analyzed data comprised 24 datasets, 12 for the “M.26” wild-type genotype and 12 for the T166 transgenic genotype of *Malus* x *domestica*. Each genotype was sampled once during February, March, April, and July and each sampling timepoint contained three independent bioreplicates (trees) at each of the timepoints. After the 24 datasets were sequenced, each was run through an optimized RNA-seq pipeline to determine statistically significant differences in gene expression between specific comparisons. The v1.0 apple reference genome and annotation used for the assembly and annotation was downloaded from Phytozome^64^.

A computational pipeline consisting of optimized tools was developed to identify differences in gene expression between the T166 and “M.26” genotypes. The pipeline consisted of: (1) read quality check using FastQC^65^; (2) data trimming using Btrim^66^; (3) reference genome indexing using HISAT2^67^; (4) alignment of trimmed reads to indexed reference genome using HISAT2^67^; (5) read count quantification using HTSeq^68^; and (6) differential expression analysis using DESeq2^69^ in R.

Four distinct comparisons of differential gene expression were considered: (1) pairwise comparisons of “M.26” vs. T166 at each timepoint; (2) separate time main effect for each genotype (“M.26” and T166; (3) pairwise comparisons of each consecutive timepoint for “M.26” and T166; and (4) interaction effect of genotype and time. The four types of comparisons provided a total of 13 comparisons, with comparison 1 being responsible for four, comparison 2 being responsible for two, comparison three being responsible for six, and comparison 4 being responsible for one. The four types of comparisons provided a comprehensive overview of the changes in gene expression corresponding to genotype (comparison 1) and time differences (comparisons 2 and 3) and which genes exhibited expression patterns that differ due to genotype over the course of the entire study (comparison 4).

The specific results for differential gene expression were determined using DESeq2, which implements a Wald test or Likelihood Ratio test to determine which genes exhibit different transcript levels within a respective comparison. The pairwise comparisons (1 and 3) utilize the Wald Test approach, which performs a parametric significance test of the selected factor level using a negative binomial distribution. Significant *p*-values result from the factor being determined as significant in the Wald test. The more complex comparisons (2 and 4) utilize a Likelihood Ratio test, which compares a full linear model considering appropriate additive and interactive effects and compares the fit against a reduced linear model with the selected factor(s) removed. Significant *p*-values result from a significant fitted improvement in the full model over the reduced model.

DESeq2 compiles a results file containing the gene ID, mean expression value, log_2_ fold-change and standard error, statistical test value, *p*-value, and adjusted *p*-value. DESeq2 adjusts the *p*-values to account for multiple testing using an FDR method. For this study, genes were considered differentially expressed if their adjusted *p*-value was below 0.05. Principal Coordinates Analysis (PCoA) was performed using Qlucore v3.2 (Qlucore, Lund, Sweden) bioinformatic software, with statistical significance set at 0.05.

### Reverse transcription-quantitative PCR (RT-qPCR)

RT-qPCR was conducted on selected genes to validate the results obtained by the RNA-Seq analysis as previously described^19^. Total RNA was diluted to 12.5 ng/μl. RT-qPCR analysis was performed using the Invitrogen SuperScript III Platinum SYBR Green One-Step RT-qPCR Kit with ROX (ThermoFisher Scientific, Waltham, MA, USA), with each reaction containing 25 ng of input RNA and 2 pmol of each primer; no-RT control reactions were included to ensure that there was no residual DNA contamination. The Applied Biosystems ViiA 7 (ThermoFisher Scientific,Waltham, MA, USA) was set to cycle as follows: cDNA synthesis at 48.0 °C for 30 min; 95.0 °C denaturation for 5 min; 40 cycles of 95.0 °C for 15 s followed by 55 °C annealing for 1 min; followed by the default ViiA 7 hold and melt curve stages. Gene-specific primers were designed using CLC Genomics Workbench (Qiagen, Valencia, CA, USA) (Supplementary Table 1). Primers were verified for specificity by using genomic DNA templates and assessing the resulting amplicon by agarose gel electrophoresis and by RT-qPCR with a subset of the sample RNA on the ViiA7. All primers produced a single band and single peak. Primer efficiency was also verified for all primer sets by RT-qPCR analysis of a standard curve constructed by serially diluting RNAs from the sample set starting at some concentration above what was used in unknown samples and ending at a concentration well below it. Three technical replicates were used for each of three biological replicates. Several endogenous reference genes (*FYPP3, LTL1*, *translation elongation factor 2*, and *CKB4*) were assessed as to the stability of their expression within the two genotypes and across timepoints^70^. *LTL1* was deemed the best overall reference gene using NormFinder software^71^. Expression levels of each of the analyzed genes were calculated using the comparative ^ΔΔ^Ct (threshold cycle) method. Data from biological replicates were used to calculate mean ± standard error (SE) expression values.

## Supplementary information

Artlip et al revised SOM

## Data Availability

The raw reads and comparisons may be obtained from the corresponding authors upon request.

## References

[1] Wisniewski M, Nassuth A, Arora R (2018). Cold hardiness in trees: a mini-review. Front. Plant Sci..

[2] Gu L (2008). The 2007 Eastern US Spring Freeze: increased cold damage in a warming wWorld?. BioSci.

[3] Wisniewski M, Artlip T, Norelli J (2016). Dealing with frost damage and climate change in tree fruit crops. New Y. Fruit. Q..

[4] Lang GA, Early JD, Martin GC, Darnell RL (1987). Endo-, para-, and ecodormancy: physiological terminology and classification for dormancy research. HortSci.

[5] Schrader J (2004). Cambial meristem dormancy in trees involves extensive remodeling of the transcriptome. Plant J..

[6] Cooke JEK, Eriksson ME, Junttila O (2012). The dynamic nature of bud dormancy in trees: environmental control and molecular mechanisms. Plant Cell Environ..

[7] Bhalerao RP, Fischer U (2016). Environmental and hormonal control of cambial stem cell dynamics. J. Exp. Bot..

[8] Tarancon C, Gonzalez-Grandio E, Oliveros JC, Nicolas M, Cubas P (2017). A conserved carbon starvation response underlies bud dormancy in woody and herbaceous species. Front. Plant Sci..

[9] Gusta LV, Wisniewski M (2013). Understanding plant cold hardiness: an opinion. Physiol. Plant..

[10] Welling A, Palva ET (2006). Molecular control of cold acclimation in trees. Physiol. Plant..

[11] Wisniewski M (2014). Genomics of cold hardiness in woody plants.. Crit. Rev. Plant Sci..

[12] Kaplan F (2007). Transcript and metabolite profiling during cold acclimation of Arabidopsis reveals an intricate relationship of cold-regulated gene expression with modifications in metabolite content. Plant J..

[13] Mizoi J, Shinozaki K, Yamaguchi-Shinozaki K (2012). AP2/ERF family transcription factors in plant abiotic stress responses. Biochim. Biophys. Acta.

[14] Vogel JT, Zarka DG, Van Buskirk HA, Fowler SG, Thomashow MF (2005). Roles of the CBF2 and ZAT12 transcription factors in configuring the low temperature transcriptome of *Arabidopsis*. Plant J..

[15] Shi Y, Ding Y, Yang S (2018). Molecular regulation of CBF signaling in cold acclimation. Trends Plant Sci..

[16] Wisniewski M, Norelli J, Bassett C, Artlip T, Macarisin D (2011). Ectopic expression of a novel peach (*Prunus persica) CBF* transcription factor in apple (*Malus x domestica*) results in short-day induced dormancy and increased cold hardiness. Planta.

[17] Heide OM, Prestrud AK (2005). Low temperature, but not photoperiod, controls growth cessation and dormancy induction and release in apple and pear. Tree Physiol..

[18] Artlip TS, Wisniewski ME, Norelli JL (2014). Field evaluation of apple overexpressing a peach *CBF* gene confirms its effect on cold hardiness, dormancy, and growth. Environ. Exp. Bot..

[19] Wisniewski M, Norelli J, Artlip T (2015). Overexpression of a peach CBF in apple: a model for understanding the integration of growth, dormancy, and cold hardiness in woody plants. Front. Plant Sci..

[20] Yordanov YS, Ma C, Strauss SH, Buscov VB (2014). *EARLY BUD-BREAK 1* (*EBB1*) is a regulator of release from seasonal dormancy in poplar trees. Proc. Natl Acad. Sci..

[21] Velasco R (2010). The genome of the domesticated apple (*Malus* x *domestica* Borkh.). Nat. Genet..

[22] Kotoda N, Wada M (2004). MdTFL1, a TFL1-like gene of apple, retards the transition from vegetative to reproductive phase in transgenic. Arabidopsis. Plant Sci..

[23] Kotoda N (2010). Molecular characterization of *FLOWERING LOCUS T-LIKE* genes of apple (*Malus* x *domestica* Borkh.). Plant Cell Physiol..

[24] Etchells JP, Mishra LS, Kumar M, Campbell L, Turner SR (2015). Wood formation in trees is increased by manipulating PXY-regulated cell division. Curr. Biol..

[25] Rinne PLH (2011). Chilling of dormant buds hyperinduces *FLOWERING LOCUS T* and recruits GA-inducible 1,3-β-glucansases to reopen signal conduits and release dormancy in *Populus*. Plant Cell.

[26] Medina J, Catala R, Salinas J (2011). The CBFs: three Arabidopsis transcription factors to cold acclimate. Plant Sci..

[27] Qin F, Shinozaki K, Yamaguchi-Shinozaki K (2011). Achievements and challenges in understanding plant abiotic stress responses and tolerance. Plant Cell Physiol..

[28] Stockinger EJ, Gilmour SJ, Thomashow MF (1997). *Arabidopsis thaliana CBF1* encodes an AP2 domain transcriptional activator that binds to the C-repeat cis-acting DNA regulatory element that stimulates in response to low temperature and water deficit. Proc. Natl Acad. Sci..

[29] Maruyama K (2004). Identification of cold-inducible downstream genes of the Arabidopsis DREB1A/CBF3 transcriptional factor using two microarray systems. Plant J..

[30] Artlip TS, Wisniewski ME, Arora R, Norelli JL (2016). An Apple rootstock overexpressing a peach CBF gene alters growth and flowering in the scion but does not impact cold hardiness or dormancy. Hortic. Res..

[31] Shim D (2014). A molecular framework for seasonal growth-dormancy regulation in perennial plants. Hortic. Res..

[32] Zohner CM, Renner SS (2015). Perception of photoperiod in individual buds of mature trees regulates leaf-out. New Phytol..

[33] Artlip, T. S. & Wisniewski, M. E. in *Handbook of Plant and Crop Physiology*, 2nd edn. (ed. Pessarakli, M.) Ch. 33 (Marcel Dekker, Inc., New York, 2001).

[34] Kerk D, Bulgrien J, Smith DW, Gribskov M (2003). Arabidopsis proteins containing similarity to the universal stress protein domain of bacteria. Plant Phys..

[35] Achard P (2008). The cold-inducible CBF1 factor-dependent signaling pathway modulates the accumulation of the growth-repressing DELLA proteins via its effect on gibberellin metabolism. Plant Cell.

[36] Niu S, Gao Q, Li Z, Chen X, Li W (2014). The role of gibberellin in the CBF1-mediated stress-response pathway. Plant Mol. Biol. Rep..

[37] Suo H (2012). Overexpression of *AtDREB1A* causes a severe dwarf phenotype by decreasing endogenous gibberellin levels in soybean [*Glycine max* (L.) Merr.]. PLoS One.

[38] Baba K (2011). Activity-dormancy transition in the cambial meristem involves stage-specific modulation of auxin response in hybrid aspen. Proc. Natl Acad. Sci. USA.

[39] Achard P, Genschik P (2009). Releasing the brakes of plant growth: how GAs shutdown DELLA proteins. J. Exp. Bot..

[40] Xu H, Liu Q, Yao T, Fu X (2014). Shedding light on integrative GA signaling. Curr. Opin. Plant Biol..

[41] Ding J, Nilsson O (2016). Molecular regulation of phenology in trees—because the seasons they are a-changin’. Curr. Opin. Plant Biol..

[42] Petterle A, Karlberg A, Bhalerao RP (2016). Daylength mediated control of seasonal growth patterns in perennial trees. Curr. Opin. Plant Biol..

[43] Preston JC, Sandve SR (2013). Adaptation to seasonality and the winter freeze. Front. Plant Sci..

[44] Tylewicz S (2018). Photoperiodic control of seasonal growth is mediated by ABA acting on cell-cell communication. Science.

[45] Li J, Niu Q, He L, Teng Y, Bai S (2018). Abscisic acid (ABA) promotes the induction and maintenance of pear (*Pyrus pyrifolia* White pear group) flower bud endodormancy. Int. J. Mol. Sci..

[46] Saito T (2013). Expression and genomic structure of the *dormancy-associated MADS box* genes *MADS13* in Japanese pears (*Pyrus pyrifolia* Nakia) that differ in their chilling requirement for endodormancy release. Tree Physiol..

[47] Wu R (2017). SVP-like MADS box genes control dormancy and budbreak in apple. Front. Plant Sci..

[48] Xing L-B (2015). Transcription profiles reveal sugar and hormone signaling pathways mediating flower induction in apple (*Malus* x *domestica* Borkh.). Plant Cell Physiol..

[49] Sun Q, Yoda K, Suzuki M, Suzuki H (2003). Vascular tissue in the stem and roots of woody plants can conduct light. J. Exp. Bot..

[50] Mimida N (2011). Apple FLOWERING LOCUS T proteins interact with transcription factors implicated in cell growth and organ development. Tree Physiol..

[51] Porto DD (2015). Transcription profiling of the chilling requirement for bud break in apples: a putative role for *FLC-like* genes. J. Exp. Bot..

[52] Kumar G (2016). Comparative phylogenetic analysis and transcriptional profiling of MADS-box gene family identified *DAM* and *FLC*-like genes in apple (*Malus* x *domestica*). Sci. Rep..

[53] Cseke LJ, Xheng J, Podila GK (2003). Characterization of *PTM5* in aspen trees: a MADS-box gene expressed during woody vascular development. Gene.

[54] Watillon B, Kettmann R, Boxus P, Burny A (1997). *Knotted1*-like homeobox genes are expressed during apple (*Malus domestica* [L.] Borkh) growth and development. Plant Mol. Biol..

[55] Groover AT (2006). The *Populus* homeobox gene *ARBORKNOX1* reveals overlapping mechanisms regulating the shoot apical meristem and the vascular cambium. Plant Mol. Biol..

[56] Du J, Mansfield SD, Groover AT (2009). The Populus homeobox gene ARBORKNOX2 regulates cell differentiation during secondary growth. Plant J..

[57] Bielenberg DG (2008). Sequencing and annotation of the *evergrowing* locus in peach (*Prunus persica* [L.] Batsch) reveals a cluster of six MADS-box transcription factors as candidate genes for regulation of terminal bud formation. Tree Genet. Genomes.

[58] Li Z, Reighard GL, Abbott AG, Bielenberg DG (2009). Dormancy-associated MADS genes from the *EVG* locus of peach (*Prunus persica* [L.] Batsch) have distinct seasonal and photoperiodic expression patterns. J. Exp. Bot..

[59] Jimenez S, Reighard GL, Bielenberg DG (2010). Gene expression of *DAM5* and *DAM6* is suppressed by chilling temperatures and inversely correlated with bud break rate. Plant Mol. Biol..

[60] Falavigna VS, Guitton B, Costes E, Andres F (2019). I want to (bud) break free: the potential role of *DAM* and *SVP*-like genes in regulating dormancy cycle in temperate fruit trees. Front. Plant Sci..

[61] Bai Y, Dougherty L, Xu K (2014). Towards an improved apple reference transcriptome using RNA‑seq. Mol. Genet. Genom..

[62] Ballester AR (2017). Transcriptomic response of resistant (PI61983 – Malus sieversii) and susceptible (‘Royal Gala’) parents of the GMAL4593 mapping population of apple to blue mold infection (Penicillium expansum).. Front. Plant Sci..

[63] Zhong S, Joung J-G, Zheng Y (2011). High-throughput Illumina strand specific RNA sequencing library preparation. Cold Spring Harb. Protoc..

[64] Goodstein DM (2011). Phytozome: a comparative platform for green plant genomics. Nucleic Acids Res..

[65] Andrews, S. FastQC: a quality control tool for high throughput sequence data. http://www.bioinformatics.babraham.ac.uk/projects/fastqc/ (2010).

[66] Kong Y (2011). Btrim: a fast, lightweight adapter and quality trimming program for next-generation sequencing technologies. Genomics.

[67] Kim D, Langmead B, Salzberg SL (2015). HISAT: a fast spliced aligner with low memory requirements. Nat. Methods.

[68] Anders S, Pyl PT, Huber W (2015). HTSeq—a Python framework to work with high-throughput sequencing data. Bioinformatics.

[69] Anders S, Huber W (2012). Differential Expression of RNA-Seq Data at the Gene Level—the DESeq Package..

[70] Bowen J, Ireland HS, Crowhurst R (2014). Selection of low-variance expressed *Malus x domestica* (apple) genes for use as quantitative PCR reference genes (housekeepers). Tree Genet. Genomes.

[71] Anderson CL, Jensen JL, Ømtoft TF (2004). Normalization of real-time quantitative reverse transcription-PCR data: a model-based variance estimation approach to identify genes suited for normalization, applied to bladder and colon cancer datasets. Cancer Res..

[72] Hwang I, Sheen J, Müller B (2012). Cytokinin signaling networks. Ann. Rev. Plant Biol..

[73] Korasick DA, Enders TA, Strader LC (2013). Auxin biosynthesis and storage forms. J. Exp. Bot..

