# Supplementary material for: Differential gene expression in non-transgenic and transgenic “M.26” apple overexpressing a peach CBF gene during the transition from eco-dormancy to bud break
Source: Hortic Res. 2019 Jul 11;6:86. doi: 10.1038/s41438-019-0168-9 (PMC6804898; doi:10.1038/s41438-019-0168-9)
Supplement: Supplementary file 1 — Artlip et al revised SOM [file 41438_2019_168_MOESM1_ESM.docx]

Artlip et al. RNA-seq of a *CBF*-overexpressing apple line

Supplementary Online Materials

Supplemental Table 1. RT-qPCR primer sequences for bioinformatics pipeline validation.

Supplemental Table 2. Highly differentially expressed stress protein genes. Genes were assigned as a member of the stress proteins with differential expression either higher (> 0) in T166 compared to ‘M.26’, or lower (< 0) in T166 compared to ‘M.26’ in one of the sampling months. Lack of data indicates expression levels were not significantly different from the base mean for that month.

Supplemental Table 3. Highly differentially expressed cell division genes. Genes were assigned as part of the cell division cycle with differential expression either higher (> 0) in T166 compared to ‘M.26’, or lower (< 0) in T166 compared to ‘M.26’ in one of the sampling months. Lack of data indicates expression levels were not significantly different from the base mean for that month.

Supplemental Table 4. Highly differentially expressed stimulatory plant growth regulator genes. Genes were assigned as part of the stimulatory plant growth regulator biosynthetic, signal transduction, or inactivation pathways with differential expression either higher (> 0) in T166 compared to ‘M.26’, or lower (< 0) in T166 compared to ‘M.26’ in one of the sampling months. Lack of data indicates expression levels were not significantly different from the base mean for that month.

Supplemental Table 5. Highly differentially expressed inhibitory plant growth regulator genes. Genes were assigned as part of the inhibitory plant growth regulator biosynthetic, signal transduction, or inactivation pathways with differential expression either higher (> 0) in T166 compared to ‘M.26’, or lower (< 0) in T166 compared to ‘M.26’ in one of the sampling months. Lack of data indicates expression levels were not significantly different from the base mean for that month.

Supplemental Table 6. Highly differentially expressed growth or dormancy genes. Genes were assigned as part of the growth or dormancy pathways with differential expression either higher (> 0) in T166 compared to ‘M.26’, or lower (< 0) in T166 compared to ‘M.26’ in one of the sampling months. Lack of data indicates expression levels were not significantly different from the base mean for that month.

Supplemental Table 7. Highly differentially expressed plasmodesmata hypothesis genes. Genes were assigned as part of the plasmodesmata hypothesis with differential expression either higher (> 0) in T166 compared to ‘M.26’, or lower (< 0) in T166 compared to ‘M.26’ in one of the sampling months. Lack of data indicates expression levels were not significantly different from the base mean for that month.

Supplementary Figure 1. Top panel: qc-barplot: Bar plot showing the total number of reads for each sample. Bottom panel: qc-boxplot: Boxplots generated from the read counts showing general distribution of log_2_-transformed counts for each sample. Similar general count distributions are shown for each sample.


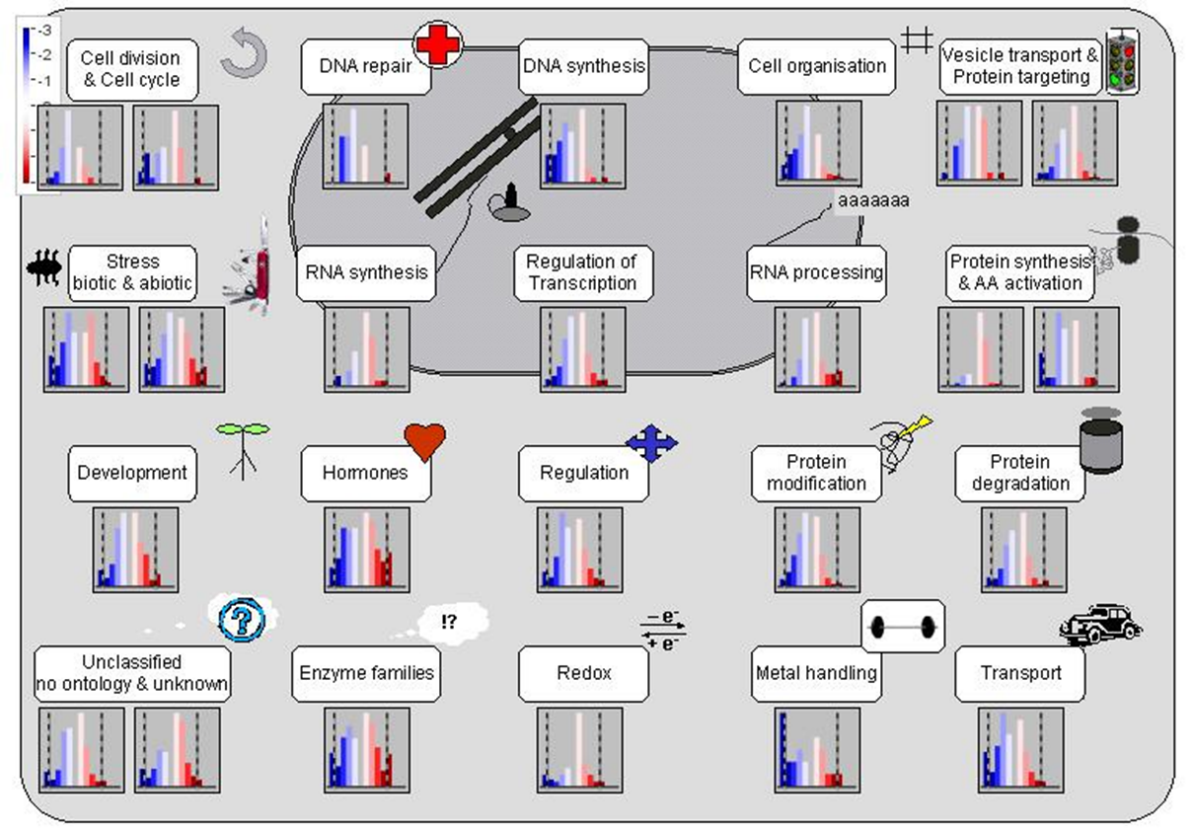


Supplemental Figure 2. Cellular Function Overview of RNA seq data comparing April bark samples from T166 and ‘M.26’ trees. The scale bar displays changes in gene expression as log2 (ratio T166/’M.26’) that were significant (FDR p value ≤ 0.01). Genes up-regulated in T166 relative to ‘M.26 are highlighted in red and down-regulated genes in T166 relative to ‘M.26’are highlighted in blue.


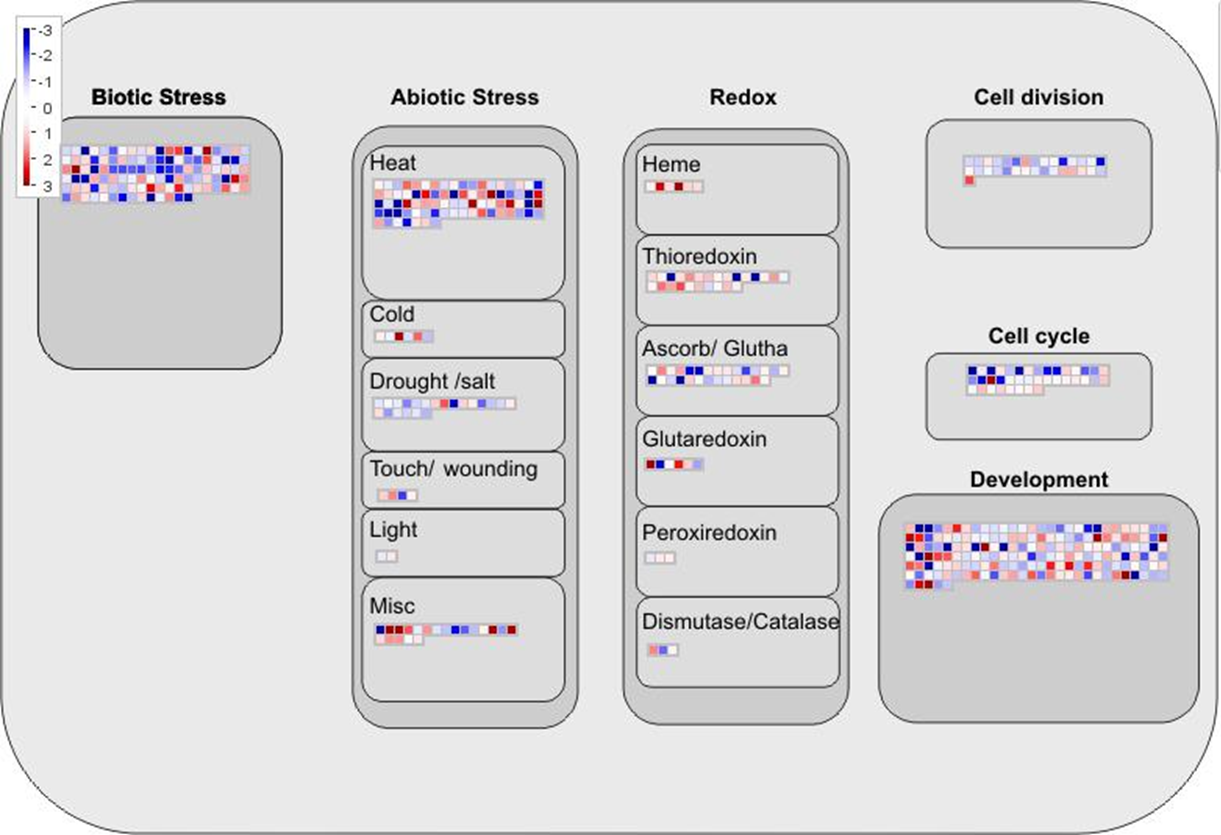


Supplemental Figure 3. Cellular Responses Overview of RNA seq data comparing April bark samples from T166 and ‘M.26’ trees. The scale bar displays changes in gene expression as log2 (ratio T166/’M.26’) that were significant (FDR p value ≤ 0.01). Genes up-regulated in T166 relative to ‘M.26 are highlighted in red and down-regulated genes in T166 relative to ‘M.26’are highlighted in blue.


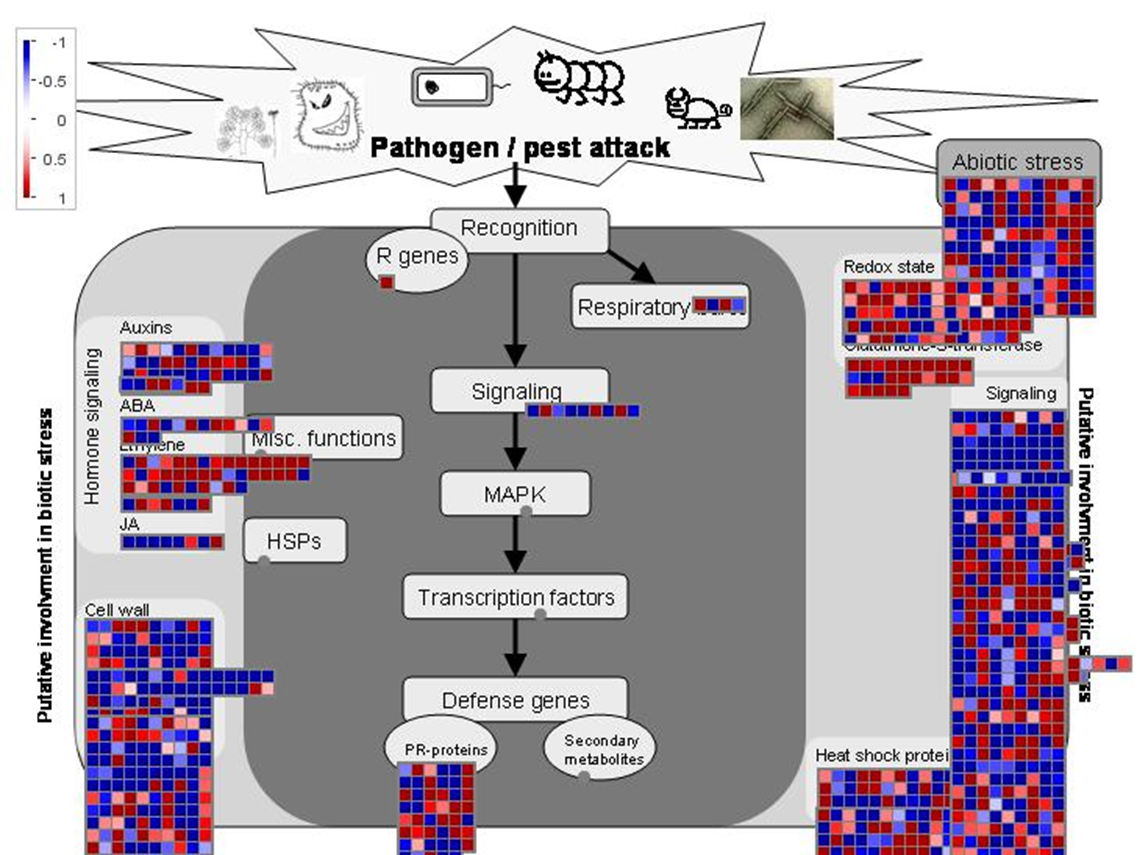


Supplemental Figure 4. Cellular Responses to Biotic Stresses from RNA seq data comparing April bark samples from T166 and ‘M.26’ trees. The scale bar displays changes in gene expression as log2 (ratio T166/’M.26’) that were significant (FDR p value ≤ 0.01). Genes up-regulated in T166 relative to ‘M.26 are highlighted in red and down-regulated genes in T166 relative to ‘M.26’are highlighted in blue.

Supplemental Figure 5. Feb-ma-plot: An MA-plot for the February comparison between M26 and T166 genotypes. Each data point represents a single gene, with the x-axis indicating log_10_ -transformed mean expression and y-axis representing the log_2_-transformed fold-change. Fold-change is based on change from M26 to T166, with higher fold-change indicating an increase in expression level from M26 to T166. Data point color indicates state of differential expression. Blue dots show genes with a significant adjusted p-value (≤ 0.05) and absolute log fold-change ≥ 1. Grey dots show genes that do not satisfy both of these properties and are therefore not considered significantly differentially expressed.

Supplemental Figure 6. Mar-ma-plot: An MA-plot for the March comparison between M26 and T166 genotypes. Each data point represents a single gene, with the x-axis indicating log_10_ -transformed mean expression and y-axis representing the log_2_-transformed fold-change. Fold-change is based on change from M26 to T166, with higher fold-change indicating an increase in expression level from M26 to T166. Data point color indicates state of differential expression. Blue dots show genes with a significant adjusted p-value (≤ 0.05) and absolute log fold-change ≥ 1. Grey dots show genes that do not satisfy both properties and are therefore not considered significantly differentially expressed.

Supplemental Figure 7. Apr-ma-plot: An MA-plot for the April comparison between M26 and T166 genotypes. Each data point represents a single gene, with the x-axis indicating log_10_ -transformed mean expression and y-axis representing the log_2_-transformed fold-change. Fold-change is based on change from M26 to T166, with higher fold-change indicating an increase in expression level from M26 to T166. Data point color indicates state of differential expression. Blue dots show genes with a significant adjusted p-value (≤ 0.05) and absolute log fold-change ≥ 1. Grey dots show genes that do not satisfy both properties and are therefore not considered significantly differentially expressed.

Supplemental Figure 8. Jul-ma-plot: An MA-plot for the July comparison between M26 and T166 genotypes. Each data point represents a single gene, with the x-axis indicating log_10_ -transformed mean expression and y-axis representing the log_2_-transformed fold-change. Fold-change is based on change from M26 to T166, with higher fold-change indicating an increase in expression level from M26 to T166. Data point color indicates state of differential expression. Blue dots show genes with a significant adjusted p-value (≤ 0.05) and absolute log fold-change ≥ 1. Grey dots show genes that do not satisfy both properties and are therefore not considered significantly differentially expressed.

Supplemental Figure 9. Feb-vol-plot: A volcano plot for the February comparison between M26 and T166 genotypes. Each data point represents a single gene, with the x-axis indicating log_2_-transformed fold-change and y-axis representing the negative log_10_-transformed adjusted p-value. Fold-change is based on change from M26 to T166, with higher fold-change indicating an increase in expression level from M26 to T166. Data point color indicates state of differential expression. Blue dots show genes with a significant adjusted p-value (≤ 0.05) and absolute log fold-change ≥ 1. Grey dots show genes that do not satisfy both properties and are therefore not considered significantly differentially expressed.

Supplemental Figure 10. Mar-vol-plot: A volcano plot for the March comparison between M26 and T166 genotypes. Each data point represents a single gene, with the x-axis indicating log_2_-transformed fold-change and y-axis representing the negative log_10_-transformed adjusted p-value. Fold-change is based on change from M26 to T166, with higher fold-change indicating an increase in expression level from M26 to T166. Data point color indicates state of differential expression. Blue dots show genes with a significant adjusted p-value (≤ 0.05) and absolute log fold-change ≥ 1. Grey dots show genes that do not satisfy both properties and are therefore not considered significantly differentially expressed.

Supplemental Figure 11. Apr-vol-plot: A volcano plot for the April comparison between M26 and T166 genotypes. Each data point represents a single gene, with the x-axis indicating log_2_-transformed fold-change and y-axis representing the negative log_10_-transformed adjusted p-value. Fold-change is based on change from M26 to T166, with higher fold-change indicating an increase in expression level from M26 to T166. Data point color indicates state of differential expression. Blue dots show genes with a significant adjusted p-value (≤ 0.05) and absolute log fold-change ≥ 1. Grey dots show genes that do not satisfy both properties and are therefore not considered significantly differentially expressed.

Supplemental Figure 12. Jul-vol-plot: A volcano plot for the July comparison between M26 and T166 genotypes. Each data point represents a single gene, with the x-axis indicating log_2_-transformed fold-change and y-axis representing the negative log_10_-transformed adjusted p-value. Fold-change is based on change from M26 to T166, with higher fold-change indicating an increase in expression level from M26 to T166. Data point color indicates state of differential expression. Blue dots show genes with a significant adjusted p-value (≤ 0.05) and absolute log fold-change ≥ 1. Grey dots show genes that do not satisfy both properties and are therefore not considered significantly differentially expressed.
